# Reduced mitochondrial pyruvate carrier expression in hearts with heart failure and reduced ejection fraction patients: ischemic vs. non-ischemic origin

**DOI:** 10.3389/fcvm.2024.1349417

**Published:** 2024-03-08

**Authors:** Paula Lopez-Vazquez, Mariana Fernandez-Caggiano, Eduardo Barge-Caballero, Gonzalo Barge-Caballero, David Couto-Mallon, Zulaika Grille-Cancela, Paula Blanco-Canosa, Maria J. Paniagua-Martin, Daniel Enriquez-Vazquez, Jose M. Vazquez-Rodriguez, Nieves Domenech, Maria G. Crespo-Leiro

**Affiliations:** ^1^ Servicio de Cardiología, Instituto de Investigación Biomédica de A Coruña (INIBIC), Complexo Hospitalario Universitario de A Coruña (CHUAC), Sergas, Universidade da Coruña (UDC), A Coruña, Spain; ^2^ Centro de Investigación Biomédica en Red de Enfermedades Cardiovasculares (CIBERCV), Instituto de Salud Carlos III, Madrid, Spain; ^3^ Barts & The London School of Medicine & Dentistry, William Harvey Research Institute, Queen Mary University of London, London, England

**Keywords:** mitochondria, mitochondrial pyruvate carrier, heart failure, ischemic heart, idiopathic dilated cardiomyopathy

## Abstract

**Introduction and objectives:**

Mitochondrial pyruvate carrier (MPC) mediates the entry of pyruvate into mitochondria, determining whether pyruvate is incorporated into the Krebs cycle or metabolized in the cytosol. In heart failure (HF), a large amount of pyruvate is metabolized to lactate in the cytosol rather than being oxidized inside the mitochondria. Thus, MPC activity or expression might play a key role in the fate of pyruvate during HF. The purpose of this work was to study the levels of the two subunits of this carrier, named MPC1 and MPC2, in human hearts with HF of different etiologies.

**Methods:**

Protein and mRNA expression analyses were conducted in cardiac tissues from three donor groups: patients with HF with reduced ejection fraction (HFrEF) with ischemic cardiomyopathy (ICM) or idiopathic dilated cardiomyopathy (IDC), and donors without cardiac pathology (Control). MPC2 plasma levels were determined by ELISA.

**Results:**

Significant reductions in the levels of MPC1, MPC2, and Sirtuin 3 (SIRT3) were observed in ICM patients compared with the levels in the Control group. However, no statistically significant differences were revealed in the analysis of MPC1 and MPC2 gene expression among the groups. Interestingly, Pyruvate dehydrogenase complex (PDH) subunits expression were increased in the ICM patients. In the case of IDC patients, a significant decrease in MPC1 was observed only when compared with the Control group. Notably, plasma MPC2 levels were found to be elevated in both disease groups compared with that in the Control group.

**Conclusion:**

Decreases in MPC1 and/or MPC2 levels were detected in the cardiac tissues of HFrEF patients, with ischemic or idiopatic origen, indicating a potential reduction in mitochondrial pyruvate uptake in the heart, which could be linked to unfavorable clinical features.

## 1 Introduction

Heart failure (HF) is a chronic progressive condition in which the heart is unable to adequately deliver oxygen and nutrients through the blood to the body's tissues (1). In HF with reduced ejection fraction (HFrEF), myocardial mitochondria do not convert sufficient nutritional energy into ATP or phosphates to meet the heart's elevated energy demands, leading to contractile abnormalities and myocardial dysfunction (2).

Mitochondria produce approximately 95% of cellular adenosine triphosphate (ATP), making them essential organelles for maintaining the high-energy demands of the heart. Mitochondria serve as a source and target of free radicals, and failure of their transmembrane potential can initiate signaling cascades involved in programmed cell death or apoptosis. Mitochondria may thus be an interesting therapeutic target for the treatment of HF (3).

Normal cardiac function depends on adequate consumption of oxygen and oxidizable substrates with the ultimate goal of generating sufficient ATP via oxidative phosphorylation to meet the heart's energy demands (4). In the healthy, well-perfused heart, approximately 70%–90% of ATP is produced by the β-oxidation of fatty acids, with most of the remainder coming from glucose and lactate oxidation. However, when oxygen is restricted, as occurs during HF, a metabolic shift occurs and glycolysis becomes the primary mechanism of ATP synthesis (5). This metabolic adaptation is supposed to be beneficial because glucose oxidation is more efficient than fatty acid oxidation in terms of oxygen consumption, but the reality is that the heart remains energetically inefficient (6).

Pyruvate, the product of glycolysis, is a major substrate for oxidative metabolism and a branch point for the synthesis of glucose, lactate, fatty acids, and amino acids (7, 8). Although protein-mediated transport of pyruvate across the mitochondrial inner membrane has been known for decades, it was in 2012 when two independent research groups used genetic and bioinformatic approaches to identify the genes and protein components of the mitochondrial pyruvate carrier (MPC): MPC1 and MPC2 (9, 10). The function of MPC is of fundamental importance in establishing the metabolic programming of a cell. Since this discovery, numerous studies have been performed to analyze its regulation in both normal and disease states (11). From this research, MPC1 and MPC2 expression levels have been shown to be decreased in some types of cancer, and their low expression has been shown to correlate with poor survival (12, 13), suggesting that regulation of the MPC complex is essential for tumor cell growth. Recent reports have shown that the mitochondrial uptake of pyruvate via MPC is necessary for the regulation of blood glucose levels in the control of gluconeogenesis in models of type 2 diabetes (14). In addition, the presence of pyruvate protects against ischemia-reperfusion (I/R) damage in the brain by activating the erythropoietin-signaling pathway (15).

In recent years, our group found that surviving tissue in the peri-infarct border zone from an *in vivo* porcine I/R model exhibited increased expression of the subunits MPC1 and MPC2 (16). We also observed that inhibition of the carrier increased infarct size and worsened cardiac function in an ex vivo mouse model of I/R (17). Moreover, cardiomyocyte-restricted deletion of MPC1 (cMPC1^−/−^) and MPC2 was shown to cause the development of pathological cardiac hypertrophy, revealing that mitochondrial pyruvate utilization is essential for myocardial adaptation to stress (18). In fact, the levels of MPC were reduced in the myocardium of mice induced to fail by angiotensin II or through transverse aortic constriction and in failing human hearts with hypertrophic growth (17, 19). Furthermore, in humans, it has been described that the abundance of MPC was also reduced in individuals with HF compared with that in healthy individuals (20). Finally, myocardial recovery induced by cardiac assist devices in patients with chronic HF was found to coincide with increased myocardial expression of MPC (21).

Pyruvate is primarily oxidized to acetyl-CoA by the pyruvate dehydrogenase (PDH) complex within the mitochondria of both heart and skeletal muscle cells (22). PDH activity is regulated by pyruvate dehydrogenase phosphatases (PDP1 and PDP2) and pyruvate dehydrogenase kinases (PDK1–4) (20). A general decrease in mitochondrial metabolism has been observed in chronic decompensated HF, with a specific decrease in pyruvate oxidation (23). This is thought to be due to decreased PDH activity caused by increased PDH kinase expression, which inactivates PDH (24), but decreased MPC abundance or activity may also be involved.

It has been revealed that MPC activity can be post-translationally regulated. In fact, recent studies have suggested that sirtuin 3 (SIRT3), a deacetylase that regulates most mitochondrial lysine acetylation (25), binds to and deacetylates MPC1 in response to high glucose concentrations to enhance pyruvate transporter activity (26). Other authors have also concluded that impaired pyruvate transport activity, mediated in part by MPC2 acetylation, contributes to impaired metabolism in the diabetic heart in a mouse model (27).

Although the pathophysiology of HF is complex, the abundance or activity of MPC may act as an important mediator of HF progression (28) by regulating the extent of pyruvate oxidation within the mitochondria. In this study, we analyzed potential alterations in MPC and PDH expression in patients with HFrHF of various etiologies. We also assessed whether the level of SIRT3, a key regulator of MPC activity, is affected during HF.

## 2 Materials and methods

### 2.1 Collection of human samples

Tissues (left ventricular myocardium, LV) and plasma samples were obtained from patients with HFrEF of ischemic origin (ICM) or cardiomyopathy of non-ischemic (idiopathic) origin (IDC) from a collection of the Advanced Heart Failure and Heart Transplantation Unit of the University Hospital of A Coruna (registration number C.0000419, A Coruña, Spain). Left ventricular biopsies were obtained from explanted hearts of patients undergoing heart transplantation. HF was defined in accordance with the European Society of Cardiology (ESC) Guidelines for the diagnosis and treatment of acute and chronic HF (29).

Control tissues were obtained from donor hearts not used for transplantation and discarded with healthy myocardium provided by the Biobank of A Coruna and the Biobank of the Principality of Asturias. The Biobank/BioCruces node of the Basque Biobank provided plasma samples from healthy-control donors. All biobanks were integrated into the Spanish National Biobank Network. All samples provided by the biobanks were processed following standard operating procedures with relevant approval from ethics and scientific committees.

### 2.2 Quantitative real-time PCR (RT-PCR)

A 30 mg sample of frozen cardiac tissue was collected and RNA was extracted using TRIzol reagent (Invitrogen), in accordance with the manufacturer's protocol. Using 2 μg aliquots of RNA, cDNAs were synthesized using the NZY First-Strand cDNA Synthesis kit (NZYTech, Lisbon, Portugal), following the manufacturer's instructions.

The PCR was performed in a LightCycler 480-II Instrument (Roche, Basel, Switzerland) using TaqMan Universal Master Mix and specific Universal ProbeLibrary (UPL) probes (Roche) of each gene (MPC1, MPC2). PCR was performed in a 10 μl reaction mixture volume with 20 μM forward primer and 20 μM reverse primer for mRNA. The PCR cycling was as follows: a denaturing step of 10 min at 95°C, followed by 50 amplification cycles of 95°C for 10 s, 60°C for 30 s, and 72°C for 1 s, followed by cooling to 40°C. Analysis of the results was carried out using Qbase + version 2.5 software and the relative expression levels of genes were estimated by the 2^−ΔΔCT^ method (30) as the average value of each sample normalized to the geometric average of the housekeeping genes GAPDH, HPRT1, and ACTB, selected after doing a geNorm study. DNA primer sequences and UPL probes are described in Table 1.

**Table 1:** DNA primer sequences and UPL probes used in the Quantitative real-time PCR.

Gen	Name	Primer F (5’-3’)	Primer R (5’-3’)	UPL probe
MPC1	Mitochondrial pyruvate carrier 1	AGGATTTCCGGGACTACCTC	GGAAGACCCCAGTTGGCTAC	#57
MPC2	Mitochondrial pyruvate carrier 2	ATGCTGCCCGAGAAATTG	TGGAGCCCAGAAGAAAACTG	#62
GAPDH	Glyceraldehyde 3-phosphate dehydrogenase	AGCCACATCGCTCAGACAC	GCCCAATACGACCAAATCC	#45
HPRT1	Hypoxanthine Phosphoribosyltransferase	TGACCTTGATTTATTTTGCATACC	CGAGCAAGACGTTCAGTCCT	#22
ACTB	Actin beta	AGAGCTACGAGCTGCCT GAC	GGATGCCACAGGACTCCA	#9

F, forward; R, reverse.

### 2.3 Western blotting (Wb)

Protein extraction was carried out using Triton lysis buffer [10 mM Tris-HCl (pH 8.0), 1 mM EDTA, 1% Triton X-100, 0.1% sodium deoxycholate, 0.1% SDS, 140 mM NaCl] and the protease inhibitor P8340 (Sigma-Aldrich. St. Louis, MO, USA), and quantified using a Pierce^™^ BCA Protein Assay Kit (Thermo Scientific, Rockford, IL, USA), following the manufacturer's instructions. After extracting protein from each tissue, 30 µg of protein was loaded into each lane of a 4%–12% gradient acrylamide Bio-Rad gel using denaturing loading buffer and proteins were separated by SDS-PAGE using the Mini-Protean 3 system (Bio-Rad, Madrid, Spain), followed by transfer to PVDF membranes (Bio-Rad). Blots were incubated with the following primary antibodies diluted in PBS-Tween 5% milk overnight at 4°C: MPC1 (Cell Signaling, #14462, Danvers, MA, USA), MPC2 (Cell Signaling, #14462), PDH (Abcam, ab110416, Cambridge, UK) and SIRT3 (Santa Cruz Biotechnology, ab1104169, Santa Cruz, CA, USA). GAPDH protein (Cell Signaling, D16H11) or tubulin (Cell Signaling, D3U1W) were used as a loading control. After washing, membranes were incubated for 1 h with horseradish peroxidase-coupled anti-rabbit (Cell Signaling, #7074) or anti-mouse secondary IgG antibody (ThermoFisher, A27025) (depending on the primary antibody) in PBS-Tween 5% milk. Enhanced chemiluminescence reagent (GE Healthcare, Chicago, IL, USA) was used to detect primary antibodies bound to the blot. Densitometry was performed on the western blots using ImageJ software.

### 2.4 Enzyme-linked immunosorbent assay (ELISA)

Plasma samples were obtained by centrifuging blood with EDTA (3,000 rpm, 15 min) and stored at −80°C until needed. Plasma MPC2 levels in patients with HFrEF and healthy controls were determined using the Human Brain Protein 44 (BRP44) ELISA kit, catalog number MBS7247075 (BRP44 is an alias for human MPC2), purchased from MyBiosource (Eersel, The Netherlands). The ELISA was performed in duplicate according to the manufacturer's instructions. Briefly, the BRP44 ELISA kit uses the competitive enzyme immunoassay technique with a polyclonal anti-BRP44 antibody and a BRP44-HRP conjugate. A standard curve is generated to correlate the color intensity with the concentration of the standards. Briefly, 100 µl of samples and standards are added to each well and incubated for one hour with 50 µl of BRP44-HRP conjugate in a pre-coated plate. After incubation, the wells are decanted and washed five times. The wells are then incubated with 100 µl of substrate for the HRP enzyme. Finally, 50 µl of Stop Solution is added to stop the reaction. The color intensity is measured spectrophotometrically at 450 nm using an ELISA plate reader (Multiskan Ascent, Thermo, MA, USA). The concentration of BRP44 in each sample is interpolated from the standard curve. (Note: We found no ELISA sufficiently sensitive to measure MPC1 levels.).

### 2.5 Statistical analysis

Student's t-test for unpaired samples was used for comparisons in experiments comprising two experimental groups when the data were normally distributed and had equal variance at a given time. Meanwhile, the Mann-Whitney test was used to assess the significance of differences between groups for non-normally distributed data. To assess the significance of differences between groups, two-way ANOVA with Bonferroni correction for *post hoc* comparisons was performed. Statistical analysis was performed with GraphPad Prism v8 and RCommander v4. Differences with *p*-values ≤0.05 were considered statistically significant. Outliers were removed based on the results of QuickCalcs, an online GraphPad tool. Results are presented as the mean ± standard error of the mean (SEM).

## 3 Results

### 3.1 Clinical and demographic characteristics of the HFrEF patient population

We used LV myocardium from HFrEF patients of ischemic origin (ICM, *n* = 20, 85% male, mean age 60.95 years) or patients with HFrEF of non-ischemic origin (idiopathic, IDC, *n* = 19, 94% male, mean age 57.26 years). Non-failing donor hearts not used for transplantation were included as a Control group (*n* = 15, 60% male, mean age 55.66 years).

### 3.2 MPC1/2 protein expression is decreased in patients with ischemic or non-ischemic HFrEF, whilst MPC1/2 gene expression remains unaltered

**Figure 1:**
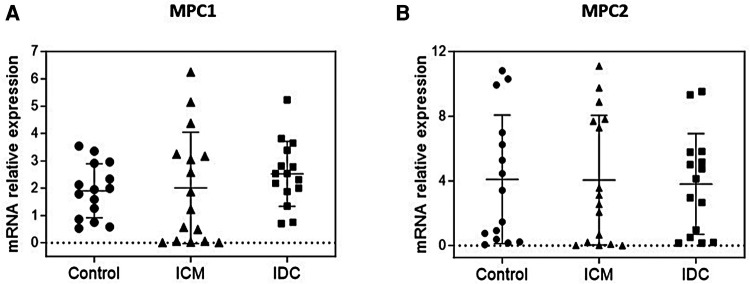
Relative gene expression levels of MPC1 and MPC2 in cardiac tissue of control group and patients with HFrEF of ischemic (ICM) or non-ischemic (idiopathic) origin (IDC). Expression levels of MPC1 (**A**) and MPC2 genes (**B**) Dots represent individuals (Control, *n* = 15; ICM, *n* = 16; IDC, *n* = 15) and mean ± standard deviation scatter cloud values are plotted.

**Figure 2:**
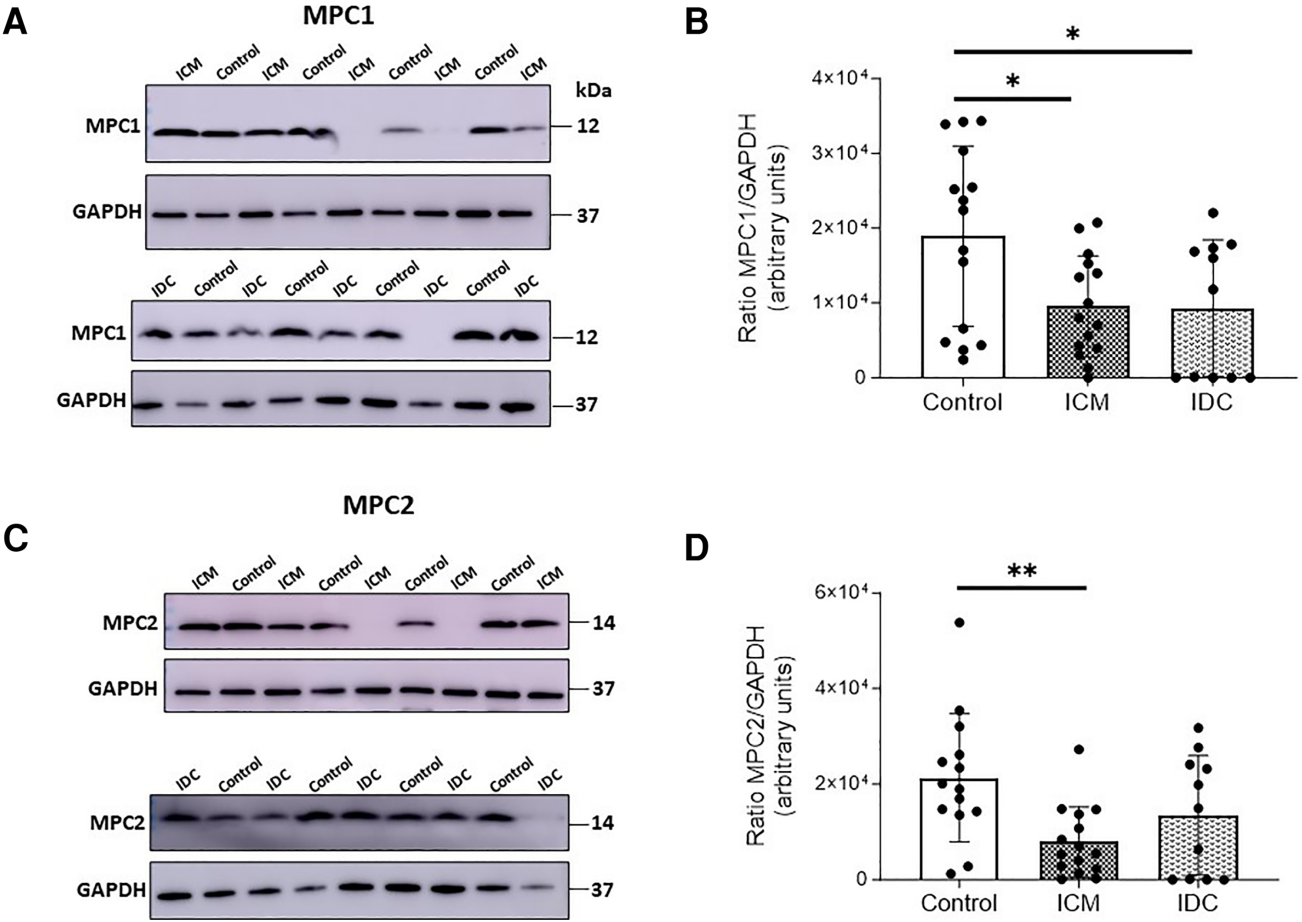
Relative protein expression levels of MPC1 and MPC2 analyzed by WB. Representative images of WB and corresponding quantitative densitometry of MPC1 (**A**) or MPC2 protein expression (**C**) from cardiac tissue of control patients and patients with HFrEF of ischemic (ICM) or non-ischemic (idiopathic) origin (IDC). Corresponding quantitative densitometry of MPC1 (**B**) or MPC2 protein expression (**D**) Protein values were normalized using GAPDH. Points represent individuals (Control, *n* = 15; ICM, *n* = 15; IDC, *n* = 11), and mean ± standard deviation scatter cloud values are plotted. *p < 0.05; **p < 0.01.

The results of the gene expression analysis showed no statistically significant difference in *MPC1* and *MPC2* expression levels in either ICM or IDC patients (Figure 1). However, protein expression analyses showed a statistically significant decrease in MPC1 expression levels in the ICM group (*p* < 0.05) and the IDC group (*p* < 0.05), compared with that in the Control group. MPC2 protein expression was significantly reduced only in the ICM group (*p* < 0.01) but not in the IDC group (*p* = 0.1475) (Figure 2).

### 3.3 Regulation of other glucose metabolism intermediates such as pyruvate dehydrogenase (PDH) and sirtuin 3 (SIRT3) in patients with ischemic or non-ischemic HFrEF

**Figure 3:**
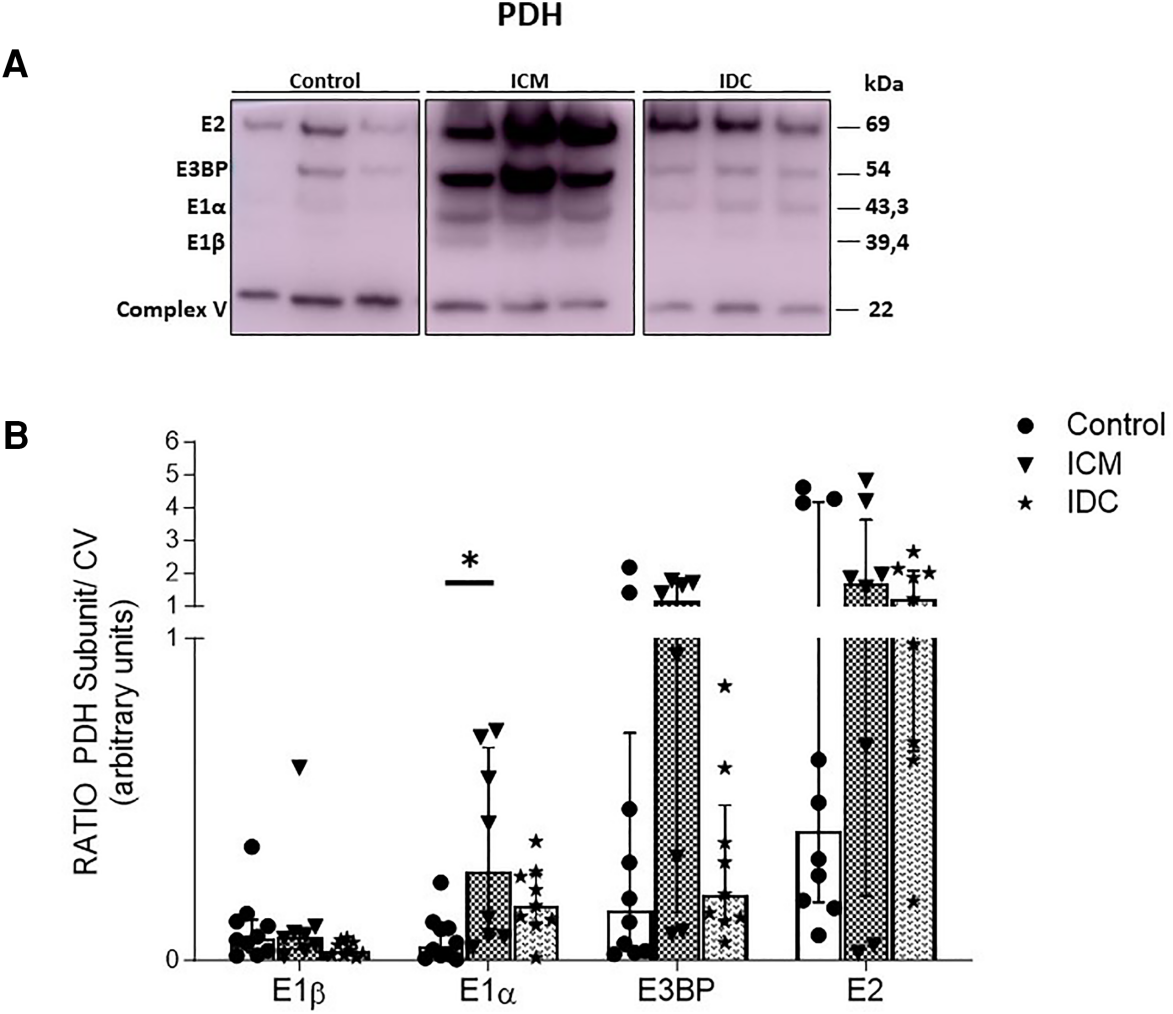
Relative protein expression levels of PDH subunits analyzed by WB. Representative images of WB of the protein expression of PDH subunits E2, E3BP, E1α, and E1β (**A**) from cardiac tissue of controls and patients with HFrEF of ischemic (ICM) or non-ischemic (idiopathic) origin (IDC). Corresponding quantitative densitometry of protein expression of the PDH subunits E2, E3BP, E1α, and E1β (**B**) Protein values were normalized using the PDH V-chain complex. Points represent individuals (Control, *n* = 10; ICM, *n* = 9; IDC, *n* = 8) and mean ± standard deviation scatter cloud values are presented. *p < 0.05.

**Figure 4:**
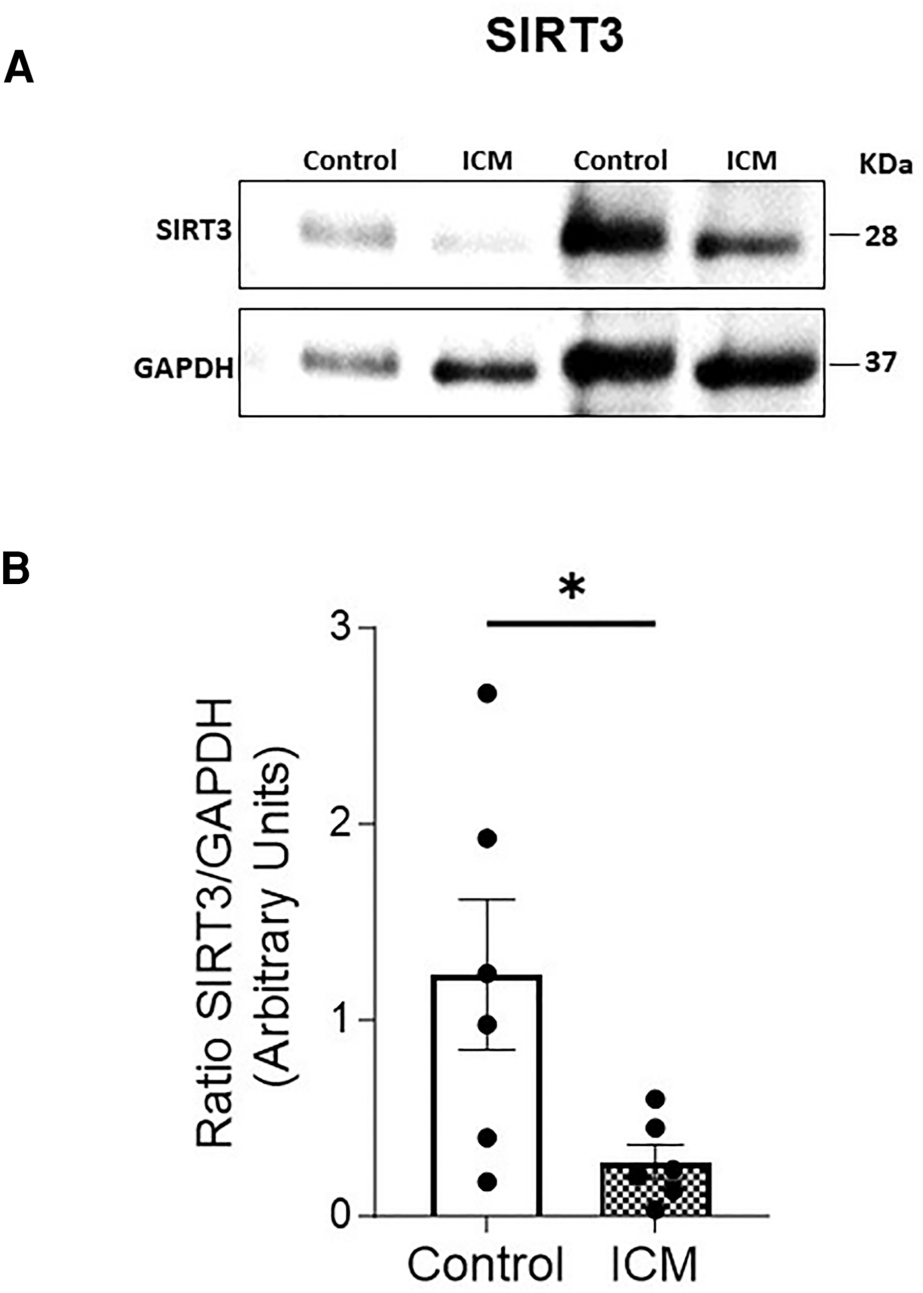
Relative protein expression levels of SIRT3 analyzed by WB. Representative images of WB of SIRT3 (**A**) from cardiac tissue of controls and patients with HFrEF of ischemic origin (ICM). Corresponding quantitative densitometry of SIRT3 protein expression (**B**) Protein values were normalized using GADPH. Points represent individuals (Control, *n* = 6; ICM, *n* = 6) and mean ± standard deviation scatter cloud values are presented. *p < 0.05; **p < 0.01.

We also wanted to analyze possible changes in the expression of other important mitochondrial molecules involved in energy production in cardiac tissue, which had been shown in previous studies to be related to the expression and regulation of the MPC complex. Therefore, we selected other molecules related to glucose metabolism, such as PDH and SIRT3, for additional analysis. Overall, the relative protein expression levels of the E2, E3BP, E1α, and E1β subunits of PDH were higher in both disease groups than in the control, but the only significant result was an increase in expression of the E1α subunit in the ICM group (*p* < 0.05) compared with that in the Control group (Figure 3). When we analyzed SIRT3 expression, the results showed a significant decrease in expression in the ICM group (*p* < 0.05) compared with that in the Control group (Figure 4) but not in the IDC group (data not shown).

### 3.4 Plasma MPC2 level is increased in patients with ischemic or non-ischemic HFrEF

**Figure 5:**
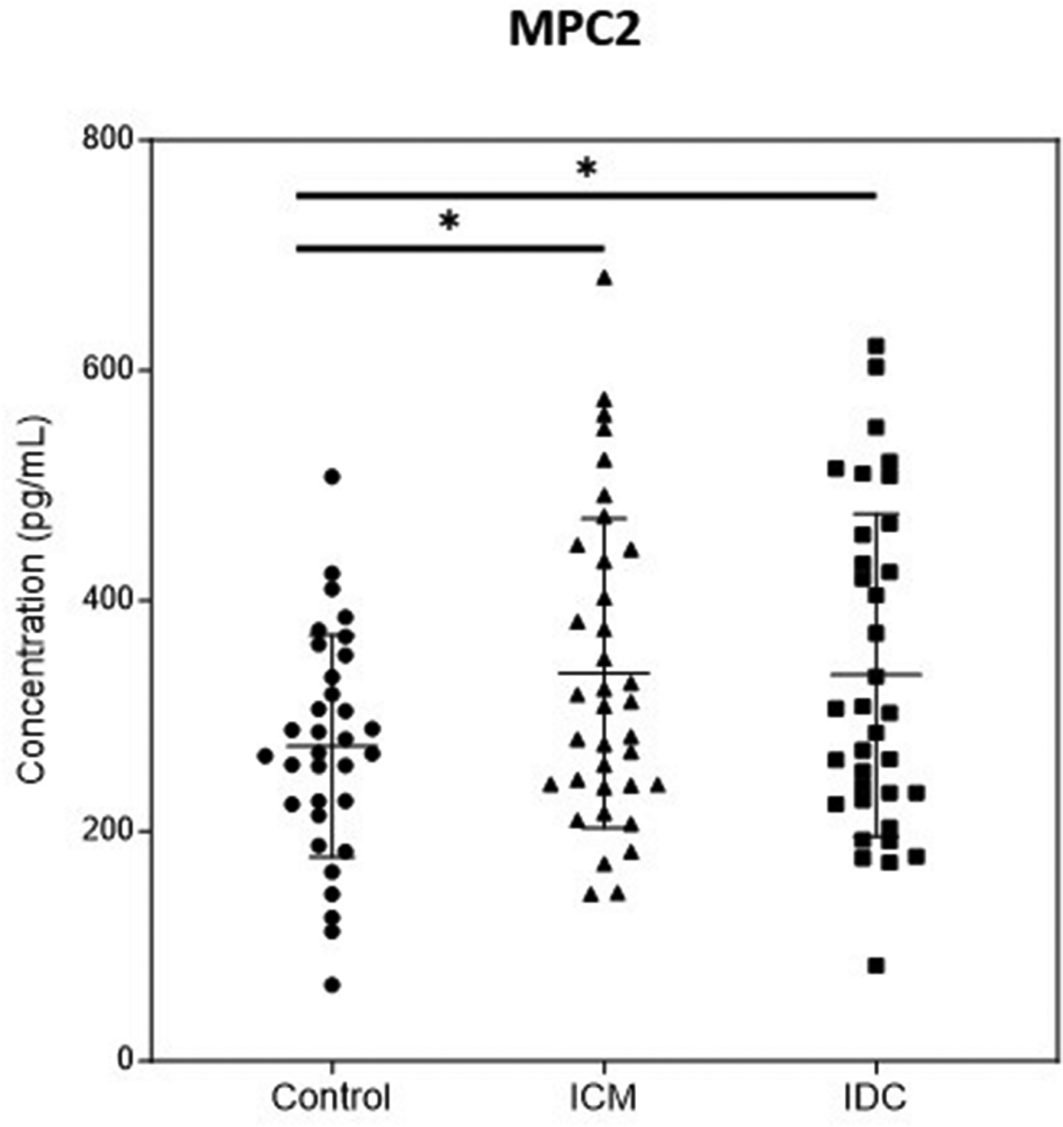
Quantification of plasma MPC2 level in control group and patients with ischemic (ICM) or non-ischemic (IDC) HFrEF. The figure illustrates protein concentrations measured by ELISA. Points represent individuals (Control, *n* = 33; ICM, *n* = 36; IDC, *n* = 35) and mean ± standard deviation scatter cloud values are presented. *p < 0.05.

Finally, we analyzed the possible presence of MPC1 and MPC2 in plasma from patients with HF to study its possible use as biomarkers. Unfortunately, we were unable to obtain results in the case of MPC1 because the obtained values were below the detection limit of the commercially available ELISA kits. However, competitive ELISA was used to assess plasma MPC2 in the Control group (*n* = 33) and in patients with HFrEF of ischemic (ICM, *n* = 36) or non-ischemic origin (IDC, *n* = 35). We found that the plasma MPC2 level was significantly higher in patients with ICM (*p* < 0.05) and patients with IDC (*p* < 0.05) than in the Control group (Figure 5).

## 4 Discussion

Mitochondria and mitochondrial proteins are promising pharmacological target candidates in the search for new molecular targets and drugs for treatments to directly improve cardiac function (31, 32). In HF, a large amount of pyruvate is metabolized to lactate in the cytosol, rather than following the mitochondrial pathway. MPC is essential for facilitating the entry of pyruvate into the mitochondria; otherwise, pyruvate is reduced to lactate within the cytoplasm (28). Against this background, the primary objective of this study was to analyze the gene and protein expression of MPC1 and MPC2 in cardiac tissue from patients with ischemic (ICM) and non-ischemic (IDC) HFrEF. The analysis of MPC1 and MPC2 gene expression showed no differences among the analyzed groups. However, protein expression analysis revealed a statistically significant decrease in the expression levels of both MPC1 and MPC2 in the ICM group compared with the Control levels. In the case of the IDC group, we found a significant difference only in MPC1 but not in MPC2 expression, which might be consistent with MPC1 activity being controlled by post-translational regulation (33).

It has been reported that the downregulation of MPC expression leads to cardiac dysfunction (17, 18). In this study, we demonstrated that failing myocardium expresses significantly lower levels of MPC1 and/or MPC2 than non-failing hearts. These results are in agreement with previous findings on the downregulation of MPC subunits in cardiac tissues from patients with HF (irrespective of the etiology) (20), or specifically in those with hypertrophic cardiomyopathy (19). Reduced MPC expression could potentially result in a diminished capacity of the mitochondria of HF patients to take up pyruvate. This effect could possibly be more pronounced in individuals with ischemic cardiomyopathy, in whom we observed lower MPC expression. A general decrease in mitochondrial metabolism has been observed in chronic decompensated HF and this is especially relevant in the context of aerobic glycolysis (34) such ischemia, however also patients with IDC have alterations in myocardial metabolism characterized by a decrease in fatty acid metabolism and an increase in myocardial glucose metabolism (35). Our results demonstrate a decrease in the levels of MPC in the pathogenesis of HF following situations that cause tissue injury, such as ischemia as in patients with ICM, but also in their absence (although to a lesser extent) as occurs in tissues of idiopathic origin in the group of patients with IDC.

PDH converts pyruvate into acetyl-CoA, which subsequently enters the Krebs cycle, a pivotal step in cellular energy production within the mitochondria. Our results demonstrated a trend towards increased expression of all PDH subunits in the ICM and IDC groups compared with the levels in the Control group, although the only significant increase was in the E1α regulatory subunit in ischemic heart. These adaptations could be explained as the ability of PDH to increase its expression to facilitate glucose metabolism in the face of a deficit in pyruvate influx produced by the decrease in MPC expression. These findings are also in agreement with previous results demonstrating that patients with end-systolic HF exhibit increased activity and expression of the subunits of the PDH complex (20).

Acetylation of lysines in MPC1 and MPC2 is a post-translational modification known to reduce the proportion of pyruvate transported into mitochondria (27). SIRT3 plays a pivotal role in this process by deacetylating MPC1, leading to an increase in pyruvate uptake into the mitochondria (26). Our findings reveal a decrease in SIRT3 expression within the ICM group compared to the levels of the control group, which could potentially result in reduced deacetylation and subsequent inactivation of MPC more specifically in the presence of ischemia.

Finally, given the downregulation of MPC subunit expression observed in cardiac tissues of patients with HFrEF, our next step was to analyze the expression levels of MPC1 and MPC2 molecules in plasma samples from these patients, in order to study their potential use as biomarkers. Unfortunately, we did not find a specific ELISA sufficiently sensitive to detect MPC1. However, quantification of MPC2 protein levels revealed that, compared with the Controls, patients with ICM and IDC had significantly higher concentrations. These molecules in plasma may have diverse origins, but the results suggest that their presence is associated with the pathology of HF. However, we can only speculate about the origin of their presence, We propose that they may stem from cardiomyocyte death induced by the pathology of the patients, but we cannot discount the possibility that they may originate from other cell types or even as a consequence of a potential immune response from the analyzed patients. Thus, the presence of such molecules could be considered an indirect biomarker of HF pathology.

In conclusion, the significant decrease in MPC1 and MPC2 levels in cardiac tissues from patients with ischemic HFrEF, and the significant decrease in MPC1 only in cardiac tissues of non-ischemic origin, may imply a reduced capacity of mitochondria to uptake pyruvate, leading to adverse clinical features in both etiologies. A major limitation of our results is that they only indicate changes in the expression of MCP1 and MCP2 in myocardial tissue in general. Despite cardiomyocytes contributing roughly 65%–80% by volume in the adult mammalian heart (36, 37), we cannot determine whether these changes are specifically related to alterations in cardiomyocytes or other types of cells. Enhancing the expression or activity of the MPC to address mitochondrial dysfunction within cardiac cells might be an effective strategy for preventing heart failure.

## Data Availability

The raw data supporting the conclusions of this article will be made available by the authors, without undue reservation.
